# Effect of an Integrated Active Lessons Programme through Playful Maths Games on Self-Concept, Self-Esteem and Social Skills in Preschool Children

**DOI:** 10.3390/bs13030260

**Published:** 2023-03-15

**Authors:** Alba Cámara-Martínez, Alberto Ruiz-Ariza, Sara Suárez-Manzano, Rosa M. Cruz-Cantero, Emilio J. Martínez-López

**Affiliations:** Department of Didactics of Musical, Plastic and Body Expression, University of Jaen, 23071 Jaén, Spain

**Keywords:** active learning, cognitive load theory, embodied learning, playful maths games, physical activity across the curriculum, physically active lesson

## Abstract

The aim of this study was to analyse the effects of an integrated active lessons programme based on playful math games, of 10 weeks’ duration (30 min/day × 2 days/week), on self-concept, self-esteem and social skills in preschool children. One hundred and ninety-four preschool children (53.6% girls) aged 53.36 ± 11.82 months were split into a control group (CG) and an experimental group (EG). The EG improved significantly in self-concept compared to the CG in boys and girls (6.8% and 7.1%, respectively). Personal and academic self-esteem levels increased significantly (8.1% and 5.5%, respectively, only in girls). Although the EG obtained significant improvements in social self-esteem and social skills compared to the beginning of the study, these results were not found in comparison with the CG. The introduction of active lessons based on playful maths games within the classroom is recommended as support for the improvement in self-concept, self-esteem and social skills in early childhood education.

## 1. Introduction

Self-concept is defined as a construct constituted by academic, personal, emotional, social, physical and family dimensions, with reference to the general objective or subjective self-perception that people have of themselves based on experiences with others and on the opinions that they have about their own behaviours [1,2]. Self-concept is key in the construction of a global positive image and is a motivational source that directly affects conduct, learning and expectations in children [3]. Moreover, it is a central and decisive factor in the development of the personality and basic to the construction of the individual’s identity [1]. In fact, some authors indicate that self-concept is related to their own perception of their place in the world and to the language and the identification of influential people [2].

Self-esteem is a variable that is closely related to self-concept; it is the way in which a person perceives and evaluates themselves in experiential and environmental contexts, based on judgments of their worth and on feelings of their own appreciation [4]. Self-esteem is widely conceived as a positive attitude required for normal development, essential for a child’s personality, and it serves an identity maintenance function. It is composed of a multidimensional set of six areas (corporal, interpersonal, educational, emotional, family and control of the context), which fluctuate due to internal and external factors, such as mood changes [3]. It is shown that low self-esteem predicts poor health, unhealthy behaviours and low values of mental status in youths, such as anxiety, depression, eating problems, criminal behaviour, suicidal thoughts and limited economic projections in adulthood [4].

Taken together, self-concept and self-esteem have a wide impact on children’s mental well-being, behaviour, academic performance and health in general [4] and are very important for achieving adequate social psychology and developing positive social skills from an early age [5]. These last characteristics are composed of a set of learned behaviours that allow the subject to solve problems and relate to others in individual or interpersonal contexts, expressing emotions, attitudes, desires and opinions appropriately [6]. It has been shown that good social skills predict good academic performance in preschool children [7]. In childhood, social skills are closely linked to peer groups and leadership qualities, especially with family members, in settings where new opportunities arise that enable the learning of guidelines, norms and prohibitions, and the learning of new decisive behaviours for their future development. The educational system represents one of the factors responsible for promoting social competencies from the age of 3 years [8].

The development of self-concept, self-esteem and social skills in the educational context is important for the integral academic and personal development of children from early stages [4,7]. Thus, it is important to determine effective interventions for improving these variables from the preschool period because it is the stage where the first traits of personality and social relationships are developed [2,4]. Nevertheless, the daily implementation of tasks focused on improving self-concept, self-esteem or social skills is scarce or is not always successful [9].

Currently, students remain individually distributed and in static positions in class, hindering the construction of self-concept and different factors of self-esteem or hindering the collaboration among classmates during the teaching–learning process, which often makes the development of self-perceptions and social aspects difficult [7]. On the other hand, there are studies advocating the development of these skills in the education context, such as the one by Lai et al. [10] that claims that the cognitive abilities of children between 4 and 9 years old can be developed through games, or the one from Rodríguez-Nielsen et al. [11] who assert that learning environments are an active methodology to promote physical activity and the cognitive development of children in early childhood education. To positively affect these variables in schools, active methodologies and group interaction are necessities [12,13]. For example, interventions based on physical activity are associated with increased self-concept, self-esteem and social skills in children [14]. However, two different meta-analyses suggest that the intervention of physical activity alone had a small effect size [4] and that the interventions mixing physical and cognitive tasks provided a large magnitude of effect size (*d* = 1.12) on school variables [15]. Therefore, a current active educational methodology has emerged, integrating physical movement with the learning of academic content through integrated active lessons involving physically active games about the different subjects. For instance, children must move, jump or play different games around the class while they simultaneously learn aspects of geography [16], sciences [9] or mathematics [17]. A systematic review by [18] has confirmed that physically active academic classes not only improve the physical activity levels of school-age children but also their academic and cognitive performance, concentration and organization skills, and time spent on homework. Similarly, gross motor tasks and team sports seem more effective academically than fine motor tasks and individual sports [19].

Nevertheless, to date, these studies have solely been targeted at academic variables and conducted mostly on primary schoolchildren. In 2008, Trost et al. [20] launched the ‘move and learn’ programme, incorporating movements into the preschool curriculum, and highlighting subjects such as maths. They showed that children were more physically active and presented more motivation and enthusiasm towards learning tasks. In this line, Quinto and Klausen [21] ascertained that the involvement of games in academic tasks favours learning by implying higher levels of intrinsic motivation and less academic stress. However, the impact of this method on important aspects of the integral development of youth from the preschool stage, such as self-concept, self-esteem and social skills, is unknown. Previous studies by Erwin et al. [22] analysed the promotion of children’s health through physically active maths classes. These authors justified that maths is one of the more important subjects in early childhood. Perhaps there is an association with students who are better at mathematics from the earliest school years being more admired by their peers and more generally successful and therefore having better self-esteem and self-concept [23,24]. Finally, according to Fernández-Bustos et al. [25], it is also important to explore the differential effects according to sex when these kinds of variables are analysed. Although some studies found no differences [26], others found positive relationships only in girls [27]. This may be because of genetic and contextual factors, or because girls seem to be more inactive from an early age [28], receiving more positive effects from physical intervention than boys. 

Based on all the above reasoning, the research question was: could an integrated programme of active classes based on playful maths games (30 min/day × 2 days/week × 10 weeks) improve self-concept, self-esteem and social skills in preschool children? It was hypothesised that integrated active lessons with playful games would increase the levels of self-concept, that is, the scores in the dimensions of corporal, personal, academic, social and family self-esteem and social skills, in comparison with another group that would not receive this intervention programme (carrying out traditional and static learning methodologies). It was also hypothesised that the effects would be higher in girls than in boys.

## 2. Materials and Methods

### 2.1. General Background

This is a longitudinal quantitative pre-post study with a control group (CG) and an experimental group (EG) that performed 30 min of integrated active lessons (2 days/week over 10 weeks). Twelve classrooms from two preschool centres in the south of Spain took part in this research, six classrooms were assigned as the EG and six as the CG. The EG and CG groups were equal and randomised within each centre.

### 2.2. Participants

**Figure 1:**
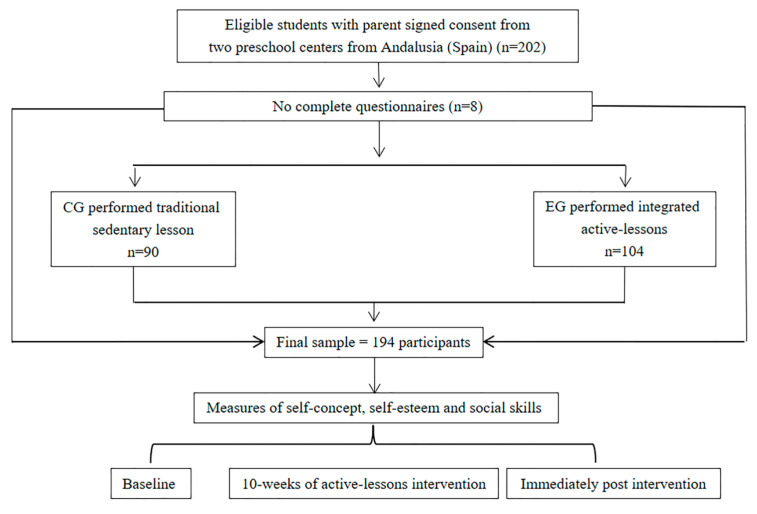
Flow of participants.

A sample of 194 preschool children aged 3 to 6 years (53.36 ± 11.82 months) participated in this study; 90 were boys (46.4%) and 104 were girls (53.6%). The sample was non-random and recruited by convenience; it had complete class groups and was biased by educational level. The structure used for group formation and intervention characteristics is shown in Figure 1.

### 2.3. Instruments and Procedures

#### 2.3.1. Self-Concept

For this variable, we used the ‘Percepción del Autoconcepto Infantil (PAI)’ test [2]. PAI is a test applied to individuals with a broad scope of use for people aged between 3 and 6 years and consists of 34 items with pictures. In each item, there is a scene with a group of children in different situations who could be classified as showing a positive or negative self-concept. In each scene, children must mark with an ‘X’ where they think they are reflected. For the child’s assessment, they will be given a score of 1 (negative self-concept) or 2 (positive self-concept). As a measure of general self-concept, the average of all items was used. The reliability was Cronbach’s α 0.832.

#### 2.3.2. Self-Esteem

The ‘Questionnaire for the Assessment of Self-esteem in children (EDINA)’, which measures self-esteem in preschool children aged 3 to 7 years, was used [5]. EDINA is composed of 21 items, divided into the following dimensions: corporal self-esteem (items: 1, 6, 16); personal self-esteem (items: 12, 17, 19, 21); academic self-esteem (items: 3, 8, 11, 13, 18); social self-esteem (items: 4, 9, 14); and family self-esteem (items: 5, 7, 10, 15, 20). Each item allows subjects to answer in three different ways: ‘yes’, ‘sometimes’ or ‘no’ (corresponding to a ‘smiling’, ‘serious’ or ‘sad face’, respectively). The reliability was Cronbach’s α 0.803.

#### 2.3.3. Social Skills in Preschool Children

The ‘Social Skills Scale for preschool children’ was used [6]. This scale aimed to assess aspects related to social interaction (among peers, prosocial manifestations, exploration of norms, understanding of emotions, detection of interpersonal behaviours, and the level of cooperation of the subject with their environment). This scale is answered by parents, and it is differentiated by age, with 12 items for children aged 3 to 4 years and 16 items for children aged 5 years. Regarding the score, the total score of the test was determined considering the assessment assigned to each of the response options (never: 1 point, sometimes: 2 points, frequently: 3 points). A higher value obtained in the test indicates a higher level of social skills. The reliability for 3, 4 and 5 years was Cronbach’s α 0.72, 0.77 and 0.86, respectively.

#### 2.3.4. Integrated Active Lessons Programme Based on Playful Maths Games

The EG performed an integrated active lessons programme based on playful maths games in the classroom, while the CG continued with the usual sedentary lessons (for example, children filled out different academic sheets with the same content as the EG, while remaining seated). The intervention consisted of an integrated active lessons programme based on playful math games, of 10 weeks’ duration (30 min/day, 2 days/week), with the aim of physical activation, group interaction and learning while moving through dynamic and participatory motor games in class (Table 1). During the initial five minutes of each session, the participants were introduced to the contents of the session. Then, the next 30 min were used to complete the games. The game proposals were competitive, but at the same time favoured both personal improvement and behaviours of respect for the rules and classmates. Finally, the last five minutes were dedicated to calm activities and the delivery of stickers and diplomas to motivate the correct execution of the tasks. In the design of the programme, it was taken into account that the methodology was participatory, active and based on games with a highly cooperative and social component.

### 2.4. Procedure

To perform the integrated active lessons programme, each teacher received instruction in this methodology during the two weeks prior to the intervention. Teachers were assigned to each study group within their school. The teachers were instructed by the research group to carry out the programme intervention. To avoid differences between groups, in the intervention model, teachers were also supervised by members of the research team during the study weeks. The schedule to perform the programme was between 12 and 12:30 p.m. The CG continued with their traditional sedentary lessons. This study was approved by the Bioethics Committee of the University of Jaén (Jaén, Spain). The design complies with the Spanish regulations for clinical research in humans (Law 14/2007, 3 July 2007, Biomedical Research), with the regulations for private data protection (Organic Law 15/1999) and with the principles of the Declaration of Helsinki (2013 version, Brazil).

### 2.5. Data Analysis

The comparison of the continuous and categorical variables according to participation in the study (CG vs. EG) was carried out through students’ *t*-tests and χ^2^, respectively. Tests of normal distribution and homogeneity (Kolmogorov–Smirnov and Levene’s, respectively) were conducted before analysis. The repeated measures analysis of covariance (ANCOVA) two times (pre, post) × two groups (CG, EG) was used to analyse the effects of the active programme. Self-concept, self-esteem and social skills were used as dependent variables, the group was used as fixed factors, and age, BMI and the maternal educational level were used as confounders. Post hoc analysis was adjusted by Bonferroni. The effect size was computed and reported as a partial *η*^2^ value for the analysis of variance (ANOVA) evaluations. To quantify the magnitude of changes between and within groups in the dependent variables, we calculated Cohen’s *d* effect sizes. A Cohen’s *d* value ≥ 0.8 indicates a large effect size, a Cohen’s *d* value ≥ 0.5 and <0.8 indicates a medium effect size, and a Cohen’s *d* value ≥ 0.2 and <0.5 indicates a small effect size [29]. Analyses were carried out separately for each dependent variable (all the samples together and split by sex). For all the analyses, a 95% confidence level was used (*p* < 0.05). The percentage of change between groups after the integrated active lessons programme was calculated as: [(EG post-measurement − CG post-measurement)/CG post-measurement] × 100. The analyses were completed using the statistical software package SPSS (v.22 for Windows, IBM Corp., Armonk, NY, USA).

## 3. Results

### 3.1. Descriptive Analysis and Correlations

Table 2 shows the anthropometric and sociodemographic characteristics of the study sample. Participants had an average of 53.36 ± 11.82 months of age, and a BMI of 15.04 ± 2.16 kg/m^2^. As for parents’ demographic characteristics, only 1.5% were uneducated and 50% had no job. The number of months of breastfeeding was similar in both study groups (*p* = 0.347). Significant differences between CG and EG were not found in self-concept, self-esteem, or social skills in pre-measurements (all *p* > 0.05).

### 3.2. ANCOVA Analysis of 10 Weeks of Integrated Active Lessons Programme Based on Playful Maths Games on Self-Concept in Preschool Children

**Figure 2:**
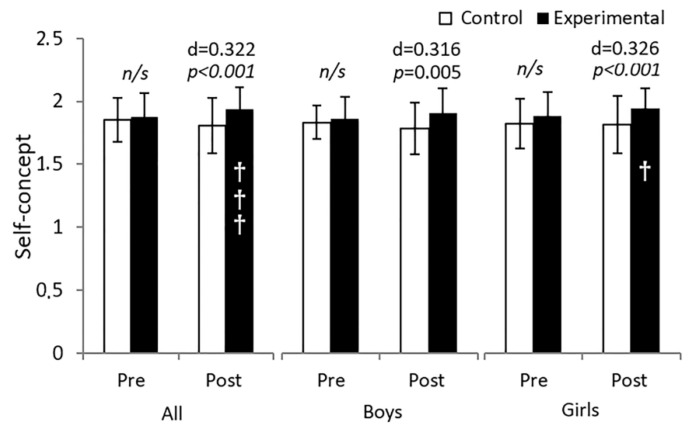
Analysis of 10 weeks of integrated active lessons programme based on playful maths games on self-conception of preschool children. † denotes *p* < 0.05; ††† denotes *p* < 0.001. *n*/*s* denotes non-significant.

Results in self-concept (Figure 2) showed a group main effect F (1, 189) = 9.541, *p* = 0.002, partial *η*^2^ = 0.048, 1 − β = 0.867 and an interaction time × group F (1, 189) = 15.697, *p* < 0.001, partial *η*^2^ = 0.777, 1 − β = 0.976. After 10 weeks the self-concept increased by 7.1% in the EG compared to the CG (1.93 ± 0.17 vs. 1.80 ± 0.21 arbitrary units (a.u.), *p* < 0.001, Cohen’s *d* = 0.322). The same analysis segmented by sex found a group main effect F (1.85) = 4.762, *p* = 0.032, partial *η*^2^ = 0.53, 1 − β = 0.578 and F (1.99) = 5.156, *p* = 0.025, partial *η*^2^ = 0.049, 1 − β = 0.614 for boys and girls, respectively, as well as an interaction time × group F (1.85) = 7.128, *p* = 0.009, partial *η*^2^ = 0.077, 1 − β = 0.753 for both boys and F (1.99) = 8.236, *p* = 0.005, partial *η*^2^ = 0.077, 1 − β = 8.236 for girls. After the intervention, the EG improved significantly compared to the CG in boys (1.93 ± 0.19 vs. 1.80 ± 0.20 = 6.8%, *p* = 0.005, Cohen’s *d* = 0.316) and in girls (1.94 ± 0.15 vs. 1.81 ± 0.22 = 7.1%, *p* < 0.001, Cohen’s *d* = 0.326).

### 3.3. ANCOVA Analysis of 10 Weeks of Integrated Active Lessons Programme Based on Playful Maths Games on Corporal, Personal, Academic, Social and Family Self-Esteem in Preschool Children

**Figure 3:**
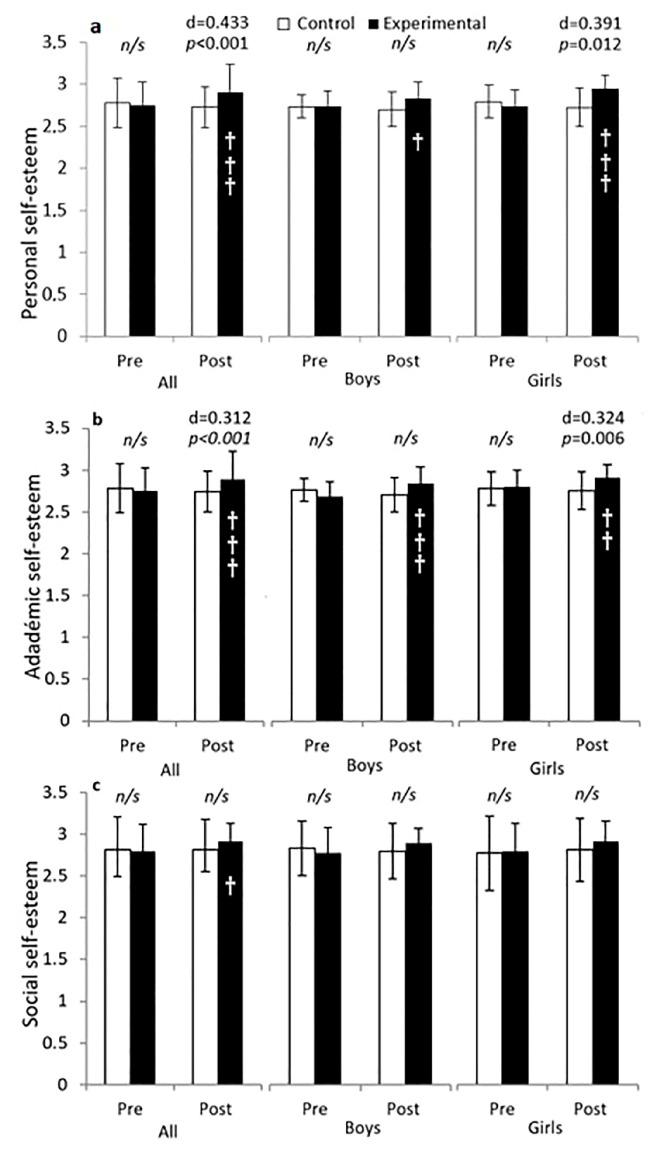
Analysis of 10 weeks of integrated active lessons programme based on playful maths games on personal, academic and social self-esteem of preschool children. (**a**) personal self-esteem; (**b**) academic self-esteem; (**c**) social self-esteem. † denotes *p* < 0.05; †† denotes *p* < 0.01; ††† denotes *p* < 0.001. *n*/*s* denotes non-significant.

The results of the analysis in the personal self-esteem factor (Figure 3a) show a group main effect F (1, 189) = 5.451, *p* = 0.021, partial *η*^2^ = 0.028, 1 − β = 0.642 and an interaction time × group F (1, 189) = 15,461, *p* < 0.001, partial *η*^2^ = 0.076, 1 − β = 0.975. After 10 weeks of the programme, personal self-esteem increased by 6.5% in the EG compared to the CG (2.90 ± 0.18 vs. 2.72 ± 0.32, *p* < 0.001; Cohen’s *d* = 0.433). In girls, the group main effect was close to significance F (1.99) = 3.584, *p* = 0.061, partial *η*^2^ = 0.035: 1 − β = 0.466. After the intervention, the EG improved by 8.1% compared to the CG (2.93 ± 0.14 vs. 2.72 ± 0.32, *p* < 0.012; Cohen’s *d* = 0.391). Boys and girls in the EG improved in their personal self-esteem compared to the pre-intervention measure (*p* = 0.029 in boys and *p* < 0.001 in girls).

The results of the analysis of the academic self-esteem factor (Figure 3b) found a group main effect F (1, 189) = 4.426, *p* = 0.037, partial *η*^2^ = 0.023, 1 − β = 0.553. After 10 weeks, the academic self-esteem had increased in the EG by 5.3% compared to the CG (2.89 ± 0.19 vs. 2.74 ± 0.26, *p* < 0.001, Cohen’s *d* = 0.312). The same analysis in girls also showed a group main effect F (1, 99) = 4.397, *p* = 0.039, partial *η*^2^ = 0.043, 1 − β = 0.546. Girls who completed the active maths lessons programme increased their academic self-esteem by 5.5% compared to the CG (2.90 ± 0.19 vs. 2.74 ± 0.27 *p* = 0.006, Cohen’s *d* = 0.324). No significant differences were found between groups in boys (*p* > 0.05). The interaction time × group was significant and similar in all cases (F (1, 189) = 14,152, *p* < 0.001, partial *η*^2^ = 0.070, 1 − β = 0.963 for the largest).

The results of the analysis of the social self-esteem factor (Figure 3c) found an interaction time × group F (1, 189) = 5.076, *p* = 0.025, partial *η*^2^ = 0.026, 1 − β = 0.611. After 10 weeks, the social self-esteem increased significantly in the EG compared to the pre-measure (post: 2.91 ± 0.21 vs. pre: 2.81 ± 0.35, *p* = 0.016). The interaction was not significant when boys and girls were analysed separately (both *p* > 0.05). No time nor group main effects were found in any of the analyses (all *p* > 0.005).

The body and family self-esteem factors (figures not shown) did not find any time, group or time × group interaction effect (all *p* > 0.05).

### 3.4. ANCOVA Analysis of the Effect of 10 Weeks of Active Maths Lessons on Social Skills

**Figure 4:**
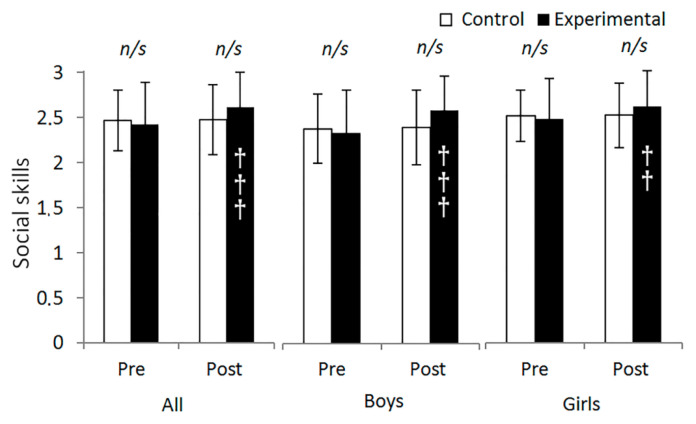
Analysis of 10 weeks of integrated active lessons programme based on playful maths games on social skills of preschool children. †† denotes *p* < 0.01; ††† denotes *p* < 0.001. *n*/*s* denotes non-significant.

The results of the analysis of social skills (Figure 4) found an interaction time × group in all the participants F (1, 189) = 13.082, *p* < 0.001, partial *η*^2^ = 0.065, 1 − β = 0.949, as well as in boys and girls F (1, 85) = 9.406, *p* = 0.003, partial *η*^2^ = 0.100, 1 − β = 0.858 and F (1, 99) = 3.879, *p* = 0.052, partial *η*^2^ = 0.038, 1 − β = 0.493, respectively. Subsequent intragroup analysis showed that the EG increased in social skills after the intervention program (post: 2.61 ± 0.38 vs. pre: 2.42 ± 0.46, *p* < 0.001). Similar results appeared in boys (post: 2.61 ± 0.38 vs. pre: 2.36 ± 0.48, *p* < 0.001) and girls (post: 2.61 ± 0.39 vs. pre: 2.47 ± 0.45, *p* = 0.008). No time or group main effects were found in any of the analyses (all *p* > 0.05).

## 4. Discussion

The present research studied the effects of an integrated active lessons programme based on playful maths games of 10 weeks’ duration (30 min/day × 2 days/week) on self-concept, self-esteem and social skills in preschool children. Results show that after 10 weeks, the EG improved significantly in self-concept compared to the CG in boys and girls (6.8% and 7.1%, respectively). Personal and academic self-esteem levels increased by 6.5% and 5.3%, respectively. Nevertheless, in line with our previous hypothesis, these differences were significant in girls but not in boys (8.1% and 5.5%, respectively). In addition, although the EG obtained significant improvements in social self-esteem and social skills compared to the beginning of the study, these results were not found in comparison with the CG. Our active lessons programme did not improve the body and family self-esteem of the participants. 

Other studies have shown that physical activity can help children achieve a positive self-concept through the improvement in physical perceptions and body satisfaction related to physical practice [25]. These authors show that BMI, body dissatisfaction or physical self-perception could be important for constructing an adequate self-concept through interventions based on physical movement. Murgui et al. [30] showed that better motor skills were linked to higher scores for self-concept, and Slutzky and Simpkins [26] also showed the positive effect of an exercise intervention on self-esteem. Some systematic reviews with metanalyses support the above results and conclude that exercise interventions improve self-concept and self-esteem in children, although they indicate that physical activity alone is less effective than integral interventions including physical activity and socio-cognitive aspects together [4].

The results of studies that have combined physical activity with mathematical calculations have shown successful results in academic performance in children and adolescents [31]. It seems that the educational use of mathematical games and problem-solving together with motor games are especially motivating for schoolchildren [31]. In this regard, our proposal of physical–academic integrated games could promote a good mood, positive emotions, well-being, motivation, interpersonal contact and good behaviour [3]. Moreover, these kinds of playful interventions could help children construct a positive image, evaluation and good feelings about themselves in an open and experiential context, essential for the growth of children’s personalities and identities and consequently beneficial to their self-concept and self-esteem [1,4].

More specifically, within the self-esteem factors, our study shows that active lessons through playful maths games increase personal and academic self-esteem levels in girls. Our intervention could produce a higher self-perception of physical ability and body image as well as an increase in playful learning, increasing personal or academic self-esteem [1,30]. Belonging to groups or working on school aspects could also be key to developing better personal and academic self-esteem [32]. These studies show that these variables have a positive effect on personal academic achievement, which is crucial for children’s lives and long-term outcomes. In contrast with our results, another study [26] showed that the relation of physical activity with self-concept and self-esteem does not vary between sexes. According to the study by [25], the positive effect of physical activity on these variables was confirmed, but they did not obtain sex differences either. In this regard, the authors of [1] indicate that, prior to adolescence, preoccupation with body image affects both genders in a similar way. In consonance with our findings, an analysis of moderation by sex in adolescents revealed that the effect of physical activity levels on global self-esteem was greater in girls than in boys [27]. Although the differential sex analysis in this kind of study in the preschool population is unknown, previous findings in some studies with adolescents and in our results could be due to the combination of biological and environmental factors [28]. For example, sex differences that could emerge due to the influence of the predominant ideal sex aesthetic from parents can be acknowledged, and it may be that preschool boys spend more time doing physical activities than girls from an early age [28]. For this reason, the improvement caused by the physical stimuli in class could positively affect personal and academic self-esteem in preschool girls more than it does in boys. Moreover, girls usually feel more positive about their academic performance [33]. For this reason, they may also have been learning more maths during the programme. In addition, because the game context allows children to learn in a less stressful context as players are allowed to make more mistakes in a game context than in an academic context, there may be a role of the game context itself beyond the physical activity component and maths component.

In addition, our intervention obtained significant improvements in social self-esteem and social skills, compared to the beginning of the study. Related to the above, diverse interventions have found a positive influence of physical activity programmes on social aspects [14]. Active lesson programmes can help children learn to work in groups, respect rules or achieve common goals [17]. Moreover, Riley et al. [34] show that children immersed in this kind of programme improved their behaviour in school by 20%. Some interventions explain that the physical–cognitive challenge of the integral games involves corporal and interpersonal processes during active playful learning, which promotes socio-cognitive benefits in the same exercise [15]. For example, jumping on different numbers on the floor in pairs or small groups requires the interpretation of visual stimuli and also requires children to make appropriate motor decisions together with their classmates, which affects variables related to social self-esteem and social skills. 

These interesting findings are in part justified by embodied cognition and the cognitive load theory. Embodied learning is produced by interactions of the body with the environment, linking out simultaneously the same cognitive processes, and contributing to the construction of higher-quality psychosocial representations [15]. The cognitive load theory is split into biologically primary development (i.e., unconscious movements) and secondary knowledge (i.e., conscious learning in schools) [35]. The cognitive load is activated during specific motor actions, providing the interrelation of cognitive and sensory-motor mechanisms, promoting social events or helping to construct the personality. In addition, active programmes can raise the level of serotonin or norepinephrine [14]. These physiological adaptations increase motivation and decrease anxiety and stress levels during academic tasks [36,37]. From this perspective, these theories could facilitate self-concept and self-esteem and might optimise social behaviour due to the playful maths games. 

Our findings support the inclusion of integrated programmes based on playful maths games and academic contents within the classroom to improve aspects related to self-concept, self-esteem and social skills in the preschool stage. Interaction among classmates and physical movement should have a key role in school curricula and serve as a tool in the educational process from an early stage. Teachers at the preschool level should be trained in this pedagogic strategy. It is recommended that teachers include at least 30 min of active lessons per day for at least 2 days/week. It is key to highlight the importance of variation within the physically active lessons in order to avoid repetitive and monotonous tasks.

## 5. Limitations and Strengths

The main limitation of the study could be that the sample was too small to be representative of the population studied. Convenience sampling is another limiting factor. The lack of previous studies on this kind of programme and its relationship with the variables studied in this population group makes it difficult to compare our results. It would be interesting to analyse the effect of these active lessons on other physical or cognitive variables. In addition, accelerometers or digital bracelets should be used in future studies to quantify the amount of physical activity during active lessons. Regarding the scale of social skills, a 3-point scale might decrease the variability, and this might be why the authors did not find a significant result. Another limitation is that we do not know whether the participants enjoyed this programme or not. A satisfaction pictorial questionnaire may have been useful. As a strength, our study provides new data and knowledge that help improve teaching and educational practice, as well as promoting active lessons from an early age to improve the psycho-social variables.

## 6. Conclusions

It is concluded that the application of active lessons programmes through playful maths games significantly increases self-concept (7.1%), and personal and academic self-esteem (6.5% and 5.3%, respectively) in preschool children. Moreover, girls are more likely to improve their personal and academic self-esteem than boys. Although there is evidence of improvement in social self-esteem and social skills, these findings have not been fully verified. The improvements in self-concept and self-esteem are verifiable, and no adverse effect has been evidenced. These results support the use of prolonged proposals based on integrated programmes with active games and academic content within the preschool classroom.

## Figures and Tables

**Table 1:** Integrated active lessons programme through playful maths games of 30 min/day of integrated active lessons (2 days/week for 10 weeks).

	Activity	Description	Materials
Week 1	Numbers song (N) Numbers with our body (N)	Children must dance the song of the numbers, imitating the movements of the dancers and making the numbers with their bodies. The teacher shows a picture of a number. In groups, children must make that number together with their own bodies on the floor.	Internet, computer and audio https://www.youtube.com/watch?v=VrZOUWNYQg0 (accessed on 1 February 2023) Pictures of numbers
Week 2	Dance of geometric figures (GF) Geometric figures with our bodies (GF)	Geometric figure images are arranged on the ground. The children must dance to the music, and when it stops, the teacher says a figure. The children look for the figure and position themselves on it. The teacher shows the image of a geometric figure. In groups, children must create that geometric figure together with their own bodies on the floor.	Geometric figure images, music Geometric figure images
Week 3	I learn to count (N) Search for numbers (N)	In groups, hopping on one leg on a number line while counting aloud. When a student reaches the last number, they must take any geometric figure and jump back with both feet. Finally, they must identify the geometric figure orally. In groups, children must run towards some cones that have images of a number at the bottom. They must lift them one by one and identify the number corresponding to their bib.	Numerical lines, geometric figures made of foam rubber Cones, images, bibs of numbers
Week 4	Twister (GF) Mini-golf (GF)	A giant map with different geometric figures. The teacher mentions actions such as ‘one hand in the triangle, one foot in the circle’. Each child must execute the teacher’s instructions at the same time, maintaining balance. Geometric figures are drawn on the ground. One child from each group should try to insert a ball, hitting it with a golf stick, into the geometric figure previously indicated by the teacher. Each child has only one try. If they fail, another team member tries until they manage to insert it into the figure.	Map with different geometric figures Balls, golf sticks, chalk or duct tape to draw the geometric figures
Week 5	We draw on the back (N and GF) Angry geometric figures (N and GF)	In pairs, one child stands on their back and the other draws the numbers and geometric figures taught by the teacher through images with their finger. The partner must guess them by enunciating their names orally. In groups, each child picks up a card that has a number and a geometric figure on it. Next, they must throw a ball and knock down the number of geometric figures that their card shows. The figures are arranged at different heights.	Numbers and geometric figures images Geometric figure and number cards, balls, foam geometric figures, toy building blocks to set different heights
Week 6	Crazy bowling (N) Domino (N)	In groups, each child throws a ball into a set of skittles. Then they walk up to them, count the knocked-down skittles and write their names on the blackboard. Groups of 10. Each child has a bib with a number. Everyone will run around the classroom. The teacher announces a number. The children with that number run to the area where the dominoes are placed and take their number.	Balls, bowling pins, chalk, blackboard Numbers, dominoes
Week 7	Bingo (N) Relays (N and GF)	Each child has a card with numbers and actions written on it. The teacher takes out numbered balls, shows them and says the number. Children who have that number on the card will put a sticker on it, stand up and perform the action shown as many times as the number indicates. In groups, a child from each group runs to the end of the classroom, picks up an image in which a number and a geometric figure appear. They leave it and jump from hoop to hoop with both feet until they reach the place where there are different geometric figures. They then take the correct number of figures and return by jumping the hoops with one leg.	Bingo cards, stickers, numbered balls Images of numbers and geometric figures, hoops, geometric foam rubber figures
Week 8	Talking phone (N and GF) Diana of numbers (N)	In groups, the children line up. Teachers give an instruction in the ear of the first child such as ‘Find two square toys’. Each child will relay the instruction to their companions in a chain until the last one receives it and looks for the objects of the instruction. Several targets are placed on the wall. Children must throw the ball towards the number that the teacher says. In the end, they will take the number of stickers corresponding to the number reached by the ball.	Toys and objects of different geometric figures Stickers, targets, balls
Week 9	Pac-Man (N and GF) Simon says (N)	Each child wears a bib with a geometric figure. In a giant labyrinth made with chalk, they must run or walk away from a child who has to catch them. In turn, they will have to collect the maximum possible number of geometric figures (corresponding to their bibs) that are found along the way. Whoever gets caught keeps it. When there are no geometric figures left in the labyrinth, each child checks if it corresponds to their number and proceeds to count them. Children are distributed around the classroom. The teacher gives instructions to the order of ‘Simon says’, which they must fulfil. For example, Simon says to clap four times and Simon says to jump one time. If the teacher’s order is not preceded by ‘Simon says’, the children should not follow it.	Chalk, geometric figures, numbers, geometric foam shapes Without materials
Week 10	The goose (N) Parcheesi (N and GF)	On the board of the game, ‘The goose’ is drawn on a large scale. The children roll the dice and move as many squares as the dice indicate. They achieve it by jumping from box to box. Depending on the square, different actions are carried out: move back two squares; advance four squares; go around three times, etc. A large-scale Parcheesi board is drawn on the floor. Each child carries a bag containing a different amount of pictures of geometric figures. One by one, they roll a die and jump the indicated number of squares. If one child overtakes another, they steal any geometric figure image from them. At the end, they will count the total number of images and each of the geometric figures.	Dice, chalk Chalk, dice, bags, geometric figure images

Note: GF = geometric figures, N = numbers.

**Table 2:** Anthropometric and sociometric characteristics of participants. Values are presented as mean and standard deviation or percentage.

		All (*n* = 194)	CG (*n* = 90)	EG (*n* = 104)	*p*-Value
Age (months)		53.36 ± 11.82	52.13 ± 11.513	54.41 ± 12.04	0.181
Weight (kg)		17.46 ± 3.98	17.29 ± 3.58	17.61 ± 4.31	0.583
Height (m)		1.07 ± 0.07	1.06 ± 0.07	1.073 ± 0.08	0.685
BMI (kg/m^2^)		15.04 ± 2.16	15.02 ± 2.36	15.06 ± 1.98	0.898
Month of lactation		10.70 ± 10.61	11.93 ± 11.67	9.86 ± 9.83	0.347
Self-concept (1–2)		1.86 ± 0.17	1.85 ± 0.17	1.87 ± 0.18	0.339
Body self-esteem (1–3)		2.86 ± 0.28	2.87 ± 0.29	2.86 ± 0.33	0.965
Personal self-esteem (1–3)		2.76 ± 0.29	2.78 ± 0.29	2.75 ± 0.29	0.545
Academic self-esteem (1–3)		2.76 ± 0.30	2.78 ± 0.31	2.75 ± 0.30	0.551
Social self-esteem (1–3)		2.80 ± 0.34	2.81 ± 0.39	2.79 ± 0.32	0.422
Family self-esteem (1–3)		2.82 ± 0.27	2.83 ± 0.28	2.82 ± 0.27	0.673
Social skills (1–3)		2.44 ± 0.41	2.46 ± 0.33	2.42 ± 0.46	0.447
Gender *n* (%)	Boy Girl	90 (46.4) 104 (53.6)	40 (44.4) 50 (55.6)	50 (48.1) 54 (51.9)	0.613
Breastfeeding *n* (%)	Yes No	104 (53.6) 90 (46.4)	42 (46.7) 48 (53.3)	62 (59.6) 42 (40.4)	0.071
Maternal Studies: Mother’s highest level of education completed *n* (%)	Without studies Primary Secondary University	3 (1.5) 13 (6.7) 104 (53.6) 74 (38.1)	1 (1.1) 5 (5.6) 50 (55.6) 34 (37.8)	2 (1.9) 8 (7.7) 54 (51.9) 40 (38.5)	0.883
Mother’s employment situation *n* (%)	Does not work Works	97 (50.0) 97 (50.0)	43 (47.8) 47 (52.2)	54 (51.9) 50 (48.1)	0.333

Notes: BMI = body mass index.

## Data Availability

The data presented in this study are not publicly available because they are confidential and proprietary.
